# Singular Case of Malformation

**Published:** 1816-03

**Authors:** 

**Affiliations:** Surgeon


### *Singular CASE of MALFORMATION.*

By Mr. JOHNSON, Surgeon.

ON the 6th of January, of the present year, in presence of Dr. Lara, I examined the body of a boy, four years of age, in whom the following curious malformations appeared. The boy had been *blue* from birth, and generally ailing, though no distinct history of the symptoms could at the moment be procured. About a fortnight before death, he was in unusual good health, from which period till his dissolution, a bowel complaint was the prominent illness, but he was not attended by any medical practitioner.

Excepting the lips, the integuments of the face, and of several parts of the body were of a *pale* colour, though before death, the whole body was *blue*. On raising the sternum, the lungs appeared, and were sound; there were four lobes, two on each side. The pericardium and heart were situated rather more towards the *right* than the left side. The pericardium contained a little water. The right auricle was very small. The foramen ovale was open; between the two *ventricles* there was another foramen, one-third of an inch in diameter. The pulmonary artery was extremely small; the aorta enormously large.

On opening the abdomen, an immense liver presented itself, filling up the epigastric and both hypochondriac regions. On insinuating the hand between the extremities of this viscus and the ribs on each side, the *left* lobe was found much larger than the right. On searching in the usual place for the *spleen*, there was none to be seen there; the liver touched the left kidney. The spleen occupied the *right* side, just above the right kidney. The gall-bladder was small, and situated exactly in the centre of the concave surface of the liver, between its two lobes. The *large* extremity of the stomach lay in the *right* hypochondrium, the small one stretching over to the left. In the ileum were six complete intus-susceptions, at nearly equal distances, some of them an inch and a half in extent. At these points, and in some places between them, were marks of inflammation.

The communications not only between the auricles, but the *ventricles* also, account for the smallness of the pulmonary artery; very little blood indeed could have been oxigenated or decarbonated in the lungs. Perhaps the *nutrient* artery of the lungs was larger than usual: I had not time to examine it; but I remarked, that the pulmonary *veins* were small, and proportioned to the pulmonary artery. Notwithstanding this singular aberration from natural structure, the boy lived four years, and probably might have lived many more, had not the intus-susceptions put an end to his existence. It is somewhat curious, that the face and *some parts* of the body turned pale after death, while *others* retained the blue colour which had existed since his birth. Did the blood in the subcutaneous vessels become decarbonated or oxigenated after death while *quiescent* and the surface exposed to the air? or, did the face and some other parts lose the blue tinge merely from the blood forsaking the extreme vessels of those parts, in articulo mortis? This I think is more likely. No perceptible difference in the *colour* could be perceived between arterial and venous blood, in the body; it was perfectly black in both ventricles of the heart, and in both sets of vessels. It is quite evident from this circumstance, and from the anatomical structure of the heart and arteries, before described, that very little oxigenation or decarbonation could have been going on during the life of this boy; and as the *abdominal* lesions were the immediate cause of death, it is fair to infer, that a complete pulmonary change in the blood is not quite so essential to the preservation of life, as has been imagined.

I forgot to observe, that the colon in this subject was not larger in calibre than the small intestines, and that it was all along one side closely beset with indentations that rendered its channel extremely narrow. Considerable thickening and disease also existed in the *caput coli*, and the mesenteric glands were in rather a morbid state, both in respect to size and structure. Hence the puny state of health of this boy may be attributed as much, perhaps, to the bad state of the digestive organs, and enormous enlargement of the liver, as to the congenital malformation in the circulating system.

The following particulars have, since the dissection, been collected from the mother of the boy.

Till the infant was two months old, the colour was not very unnatural. At this period he was seized with a *fit*, when the face and body turned quite black; the child appeared suffocating, but never struggled in the body or extremities. This went off in half an hour, but the colour never became as before the fit—it continued blue; and the fits recurred more or less frequently during its life. Excepting upon an occasion hereafter to be mentioned, the boy rarely passed a day without one or more of these fits; and as he grew older, he fell, or threw himself down on the commencement of the attack, lying apparently without any other sign of life, than short convulsive respirations; his skin quite black, and his eyes starting from their sockets. These paroxysms would frequently last for two or three hours, after which, the child would fall asleep. He was kept at the breast eighteen months, and never tasted any other kind of food during that time.

Dentition did not take place till he was weaned; after which the appetite was frequently so voracious, that he was forced to be fed several times in the night. His bowels were irregular, and the evacuations generally depraved. He could not walk till within a month of his death, at which time he appeared in better health than at any previous period, his fits being less severe, and less frequent. When two years of age, he was seized with jaundice, and during the whole time of the yellow suffusion on the surface, (about three weeks) he was not once attacked with his usual paroxysms!

The foregoing particulars relative to the boy's health, and the anatomical deviations in his structure, are calculated to afford much matter for physiological speculation. To what were the *fits* owing? Can they be attributable to any periodical *difficulty* in the circulation? or were they owing to the effect of carbonated or unoxigenated blood on the brain, producing that universal, though temporary *suspension of action* in all the voluntary muscles; or in other words, of the *animal life*, during which, an accumulation of excitability took place, that was again expended between the paroxysms? On what principle can the suspension of the paroxysms be accounted for, during the time that the bile was obstructed in its course to the intestines, and regurgitating into the circulation? Was it purely *accidental* that the *large* extremity of the *stomach* was in the *right* side, where was also the *spleen*? Was there any connection between the enormous enlargement of the liver, and the state of the circulation? Was the slow developement of the teeth, the speech, and the power of locomotion, owing to the influence of black blood on the brain? To what were attributable the irregularity and frequent voracity of the appetite?

St. George's Square, Portsea,
January 23, 1816.

---

### PART II.

### COMPREHENSIVE ANALYTICAL REVIEW OF MEDICAL LITERATURE.

" Tros, tyriusve, *nobis* nullo discrimine agetur."

### *Dr. Parry's Elements of Pathology and Therapeutics.*

